# EPHA3 enhances macrophage autophagy and apoptosis by disrupting the mTOR signaling pathway in mice with endometriosis

**DOI:** 10.1042/BSR20182274

**Published:** 2019-07-30

**Authors:** Hongmei Xu, Yongmei Gao, Yang Shu, Yi Wang, Qingyang Shi

**Affiliations:** 1 Department of Obstetrics, The First Hospital of Jilin University, Changchun 130021, Jilin, China; 2 Department of Gynaecology and Obstetrics, The First Hospital of Jilin University, Changchun 130021, Jilin, China; 3 Centre for Reproductive Medicine, Centre for Prenatal Diagnosis, The First Hospital of Jilin University, Changchun 130021, Jilin, China

**Keywords:** Apoptosis, Autophagy, Endometriosis, EPHA3, Macrophage, mTOR signaling pathway

## Abstract

**Background:** Endometriosis is a chronic fibrotic disease characterized by agonizing pelvic pain and low fertility, mainly affecting middle-aged women. The aim of the present study is to assess the potential effects of erythropoietin-producing hepatocellular carcinoma A3 (EPHA3) on endometriosis, with emphasis on the autophagy and apoptosis of macrophages via inhibition of the mammalian target of rapamycin (mTOR) signaling pathway.

**Methods:** The mouse models of endometriosis were established followed by culturing the macrophages and macrophage transfection via the EPHA3 vector, siRNA EPHA3, and RAPA (an inhibitor of the mTOR signaling pathway). The expression of EPHA3, related factors in the mTOR signaling pathway, macrophage autophagy (autophagy-related gene 3 (Atg3), light chain 3-I (LC3-I), light chain 3-II (LC3-II) and Beclin1) and apoptosis (B-cell lymphoma-2 (bcl-2), bax and fas) were all detected and documented, respectively. The changes of autophagic lysosomes and the apoptosis of macrophages in each group following transfection were also inspected and detected.

**Results:** The results of the *in silico* analysis ascertained EPHA3 to be a candidate gene of endometriosis. After successful modeling, the uterine tissues of endometriosis mice presented with a low expression of EPHA3 and activated mTOR signaling pathway. Overexpression of EPHA3 inhibited the activation of the mTOR signaling pathway, down-regulated bcl-2 expression, up-regulated the expression of Atg3, LC3-II/LC3-I, Beclin1, bax and fas, and also promoted the autophagy and apoptosis of macrophages in endometriosis mice.

**Conclusion:** Altogether, EPHA3 could potentially promote the autophagy and apoptosis of macrophages in endometriosis via inhibition of the mTOR signaling pathway, highlighting the potential of EPHA3 as the target to treat endometriosis.

## Introduction

Endometriosis is an estrogen-mediated chronic inflammatory condition which is accompanied by the appearance of endometrial tissue outside the uterine cavity [1]. Endometriosis also shares a close association with pelvic pain and infertility [2]. Women in the age range of 15–55 years have been reported to be primarily vulnerable to endometriosis [3]. However, the prevalence rate among the general population still requires estimation because it is easy to be overlooked under medical supervision [3]. Several reproductive factors have been reported to increase the risk of endometriosis, such as early age at menarche, short menstrual cycle length, parity and current oral contraceptive use [4]. Patients suffering from endometriosis have also been recommended to undergo surgical intervention, for a temporary relief from the agonizing pain that endometriosis brings [5,6]. In recent years, accumulating evidence has highlighted the impact of several genes in relation to the formation and remission of endometriosis [7,8]. Autophagy is vital in a range of pathophysiological situations, and dysregulated autophagy is associated with various human diseases, including neurodegenerative diseases and malignancy [9]. Autophagy has been speculated to play a crucial role in protecting cells for a prolonged survival by preventing apoptosis under various metabolic stress conditions, such as hypoxia or oxidative stress [10]. Interestingly, several studies have revealed that autophagy is up-regulated in ovarian endometriomas [11,12].

Erythropoietin-producing hepatocellular carcinoma A3 (EPHA3) belongs to the EPH receptor subfamily, which is the largest family of vertebrate receptor protein tyrosine kinases [13]. Mutations in the EPHA3 gene have been highlighted in tumors, which has also been regarded as a potential therapeutic target for malignancies [14]. Existing literature has demonstrated the influence of the EPHA3 during the treatment of various human tumors, such as acute lymphoblastic leukemia, glioblastoma multiforme and prostate cancer [13,15,16]. However, the function of EPHA3 in endometriosis remains unknown due to the lack of studies primarily focusing on the efficacy of EPHA3. A previous study found that Ad-TERTp-E1A-1504 has no harmful effects on normal cells but inhibits and kills TERT- and EphA3-positive tumor cells, which is mediated through the protein kinase B/mammalian target of rapamycin (AKT/mTOR) signaling pathway via autophagy induction [17]. As a serine kinase, mTOR concerns cell survival and proliferation, which acts as an important component of two unique multi-protein signaling complexes, mTORC1 and mTORC2 [18]. Meanwhile, the phosphatidylinositol 3-kinase (PI3K)/AKT/mTOR pathway is recurrently dysregulated in human cancers through a variety of molecular aberrations [19]. This evidence has flagged the functionality of the PI3K/AKT/mTOR pathway with a significant role in the pathogenesis of endometriosis [20]. With the aforementioned information serving as the basis, it was hypothesized that the EPHA3 could inhibit macrophage autophagy and apoptosis in endometriosis, which is expected to be an important breakthrough in the treatment of endometriosis.

## Materials and methods

### Microarray-based gene expression profiling

The National Center for Biotechnology Information (NCBI) is a public platform for storing gene expression datasets, original sequences and records. The GSE5108 dataset was found by searching for datasets relevant to endometriosis, which comprised 11 endometriosis samples and 11 normal control samples. Following that, important differentially expressed genes (DEGs) were selected using the R language based on the specifications of the Bioconductor’s ‘limma’ package. At last, DEGs were annotated by the ‘annotate’ package. A value of *P*<0.05 was considered to be statistically significant.

Gene ontology (GO) analysis was conducted via the website available at http://www.webgestalt.org/. Key genes and important gene modules involved in the development of endometriosis were identified from the level of protein interaction by the protein–protein interaction (PPI) network. PPI information of the selected DEGs was obtained using a search tool (STRING) database (http://www.stringdb.org/) for retrieving the interactive genes. At last, the PPI network was constructed using the Cytoscape software. A value of *P*<0.05 was considered to be statistically significant.

### Identification and establishment of mouse models of endometriosis

**Figure 1:**
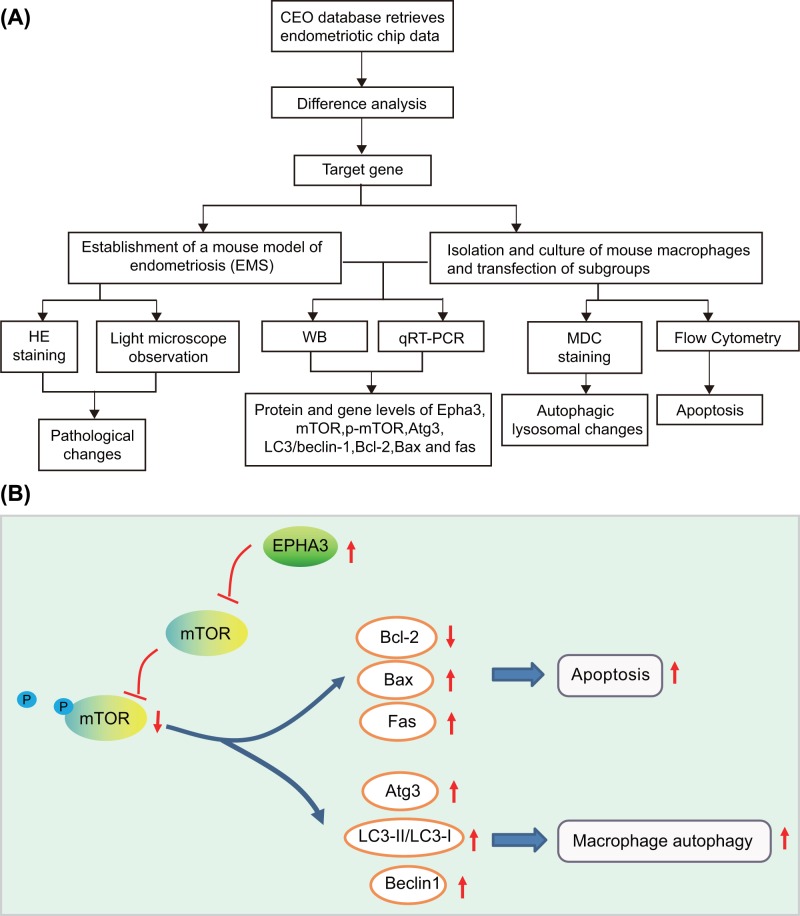
A flow chart and the mechanism of EPHA3 in endometriosis (**A**) The flow chart illustrating the experimental procedures employed in the present study. (**B**) The schematic diagram showing the effect of EPHA3 in endometriosis.

Fifty-five specific-pathogen free (SPF) female BALB/c mice were a part of the present study (weight: 18–25 g; estrous cycle: 4–5 days; Fuzhou Institute of Experimental Animals, Fuzhou, Fujian, China). According to the estrous cycle, five mice were housed in one cage at 21–23°C with a relative humidity of 60–75% for a 12-h day/light cycle with free access to normal water and food. These mice were allocated into the endometriosis group (*n*=20), sham group (*n*=15), normal group (*n*=15) and donor group (*n*=5). Before initiation for any therapeutic intervention, each mouse was anesthetized with an intraperitoneal injection of 1.6 mg thiopental sodium dissolved in 0.65 ml sterilized saline. Then five mice in the donor group were killed via the CO_2_ method [21], and fixed in a supine position. After sterilization using alcohol, their abdominal cavities were incised along the ventrimeson. After the removal of the uteruses, the myometrium and adjacent adipose tissues were removed and cultured in a Petri dish containing 10 ml sterilized saline (37°C). Following the removal of the blood, myometrium and fat fragments, the uteruses was divided into two sections. The uterine horn was placed in a separate Petri dish and the finely chopped pieces of 1 mm^3^ blocks were dissolved in 1 ml of sterilized saline (37°C). After sterilization using alcohol, the 20 mice in the endometriosis group were intraperitoneally injected with 1 ml normal saline containing endometrial fragments [22]. The selected injection sites were 0.5 cm away from the left lower quadrant below the umbilicus. Fifteen out of the seventeen modeled mice were chosen for subsequent experiments. Fifteen mice in the sham group were intraperitoneally injected with 1 ml pure normal saline, and the entire procedure was completed within 5 min under aseptic conditions. The mice in the normal group underwent no treatment. The flowchart of the experiment design is shown in Figure 1A.

### Isolation and culture of mouse macrophages and extraction of endometrial tissues

On the 21st day after modeling, five mice in each group were randomly selected and killed following the CO_2_ approach after sufficient anesthesia. Next, the mice were immersed in 75% alcohol for 1 min. After drying, the mice were intraperitoneally injected with 5 ml D-Hank’s solution. The peritoneal fluid and the hemolysis of the ris-NH_4_Cl solution were suctioned through repeated operations. Subsequently, the abdominal suspension was collected at 716×***g*** for 5 min. Following the washing period, the cell concentration was adjusted to 5 × 10^5^ cells/ml using Roswell Park Memorial Institute-1640 medium. The cell suspension was then incubated at 37°C in a 5% CO_2_ incubator for 2 h. The un-adherent cells were removed following three rinses with phosphate buffer saline (PBS) while the adherent macrophages were cultured in an incubator. The abdominal cavities of the mice were exposed on a sterilized workbench for observation of the morphology of the uterine ectopic lesions via visual inspection. The endometrial tissues were then acquired separately, from which one segment was used for Hematoxylin–Eosin (HE) staining and microscopic examination to assess the model establishment, and the remaining segments were frozen in liquid nitrogen at −70°C for further use.

### HE staining

Initially, small pieces of ectopic endometrial tissue specimens fixed using 4% paraformaldehyde solution (WH1011, Shanghai Weiao Biotechnology Co., Ltd., Shanghai, China) were dehydrated using gradient alcohol of different concentrations (70, 80, 90, 95 and 100%, respectively; 5 min each time), and cleared twice with xylene (10 min each time). After being immersed in wax and embedded with paraffin, the tissue specimens were cut into 4-μm-thick sections and heated at 60°C for 1 h, followed by xylene dewaxing and gradient alcohol dehydration sequentially. Subsequently, the sections were stained using Hematoxylin (BIO-0129, Shanghai Walan Biotechnology Co., Ltd., Shanghai, China) for 10 min, differentiated with 1% hydrochloric acid alcohol for 20 s, and then finally treated with 1% ammonia for 30 s. After a round of staining with Eosin for 3 min, the sections were then dehydrated in a conventional manner using gradient ethanol (2 min each time), cleared twice with xylene (5 min each time), and sealed with neutral gum (YS-3766, Shanghai Yuanmu Biotechnology Co., Ltd., Shanghai, China). Finally, the sections were observed under a 40-fold ordinary optical microscope (Olympus Optical Co., Ltd., Tokyo, Japan) for pathological changes in the ectopic lesions.

### Trypan Blue staining

A combination of 0.5 ml macrophage suspension and 0.4% Trypan Blue staining solution was added into an Eppendorf (EP) tube. After being mixed, a drop of the prepared cell suspension was added into the neubauer hemocytometer. After 2–3 min, upon sinking, the counting plate was placed under a microscope. The number of dead and living cells was counted within 3 min, respectively. The viability was expressed as the percentage of living cells in the counted cells.

### Cell grouping and transfection

The primary macrophages were allocated into a normal group (primary macrophages of normal mice without any sequence transfection) and other six groups (primary macrophages of endometriosis mice), which were the blank group (without any sequence transfection), the negative control (NC) group (transfected with empty vector plasmid), the EPHA3 vector group (transfected with overexpressed EPHA3 plasmid), the si-EPHA3 group (transfected with si-EPHA3), the RAPA group (added with the mTOR signaling pathway inhibitor) and the EPHA3 vector + RAPA group (transfected with overexpressed EPHA3 plasmid and added with the mTOR signaling pathway inhibitor). Primary macrophages in the logarithmic growth phase from the endometriosis mice were inoculated into a six-well plate. When the cell density reached 30–50%, the cells were transfected according to the instructions of Lipofectamine 2000 (Invitrogen, Carlsbad, CA, U.S.A.).

### RNA isolation and quantitation

The endometrial tissues and cells from the BALB/c mice of each group were extracted, and the total RNA was extracted in strict accordance with the provided instructions of an ultrapure RNA extraction kit (Qiagen, Hilden, Germany). At the tissue level, the expression of EPHA3 and mTOR were determined. The primers of EPHA3, mTOR, autophagy-related gene 3 (Atg3), light chain 3 (LC3), Beclin1, bax, fas and B-cell lymphoma*-*2 (bcl-2) were synthesized by Aoke Biotechnology Co., Ltd. (Zhenjiang, Jiangsu, China) (Table 1). Next, the RNA template, Primer Mix, dNTP Mix, DTT, RT Buffer, HiFi-MMLV and RNase-free water were all dissolved on ice for later use. The extracted RNA (20 μl) was reverse transcribed in accordance with the instructions of TaqMan MicroRNA Assays Reverse Transcription Primer (4366596, Thermo Scientific, Waltham, MA, U.S.A.). The quantitative PCR was conducted in strict accordance with the instructions of the SYBR® Premix Ex Taq™ II Kit (RR820A, Xingzhi Biotechnology Co., Ltd., Guangzhou, Guangdong, China) on an ABI PRISM® 7300 (Prism® 7300, Shanghai Kunke Instrument Equipment Co., Ltd., Shanghai, China). Glyceraldehyde-3-phosphate dehydrogenase (GAPDH, abs830032, ABX Biotechnology Co., Ltd., Shanghai, China) was regarded as an internal reference, and the fold changes were calculated by means of relative quantitation (2^−ΔΔ*C*^_t_ method).

**Table 1:** Primer sequences

Target gene	Forward sequence (5′–3′)	Reverse sequence (5′–3′)	Size
*EPHA3*	CAGCCTTCCAACGAAGTTAATCT	TGCACACCTGGTAAGTCCTGA	142
*m-TOR*	CAGTTCGCCAGTGGACTGAAG	GCTGGTCATAGAAGCGAGTAGAC	130
*Atg3*	ACACGGTGAAGGGAAAGGC	TGGTGGACTAAGTGATCTCCAG	130
*LC3*	GACCGCTGTAAGGAGGTGC	CTTGACCAACTCGCTCATGTTA	153
*Beclin1*	ATGGAGGGGTCTAAGGCGTC	TGGGCTGTGGTAAGTAATGGA	149
*bcl-2*	ATGCCTTTGTGGAACTATATGGC	GGTATGCACCCAGAGTGATGC	120
*bax*	AGACAGGGGCCTTTTTGCTAC	AATTCGCCGGAGACACTCG	137
*fas*	GCGGGTTCGTGAAACTGATAA	GCAAAATGGGCCTCCTTGATA	61
*GAPDH*	AGGTCGGTGTGAACGGATTTG	TGTAGACCATGTAGTTGAGGTCA	95

### Western blot analysis

Tissues in each group or the macrophages at 48 h after transfection were lysed on to ice with 1 ml lysis buffer at 4°C for 30 min. After supernatant collection, a bicinchoninic acid (BCA) kit (20201ES76, Yisheng Biotechnology Co., Ltd., Shanghai, China) was used to determine the protein concentration. Following the conventional application of 12% sodium dodecyl sulfate/polyacrylamide gel electrophoresis (SDS/PAGE), the protein samples were electroblotted on to a polyvinylidene fluoride (PVDF) membrane, after which membrane blockade was performed with 5% skim milk overnight. The membranes were incubated with diluted primary antibodies to EPHA3 (1:800, ab110465), mTOR (1:2000, ab2732), p-mTOR (1:1000, ab84400), Atg3 (1:800, ab228749), LC3B (1:2000, ab192890), Beclin1 (1:750, ab62557), bcl-2 (1:1000, ab59348), bax (1:1000, ab199677) and fas (1:1000, ab15285) overnight at 4°C. The aforementioned antibodies were purchased from Abcam Inc. (Cambridge, MA, U.S.A.) except LC3B, which was purchased from Sigma–Aldrich Chemical Company (St Louis, MO, U.S.A.). The membrane was then incubated with the horseradish peroxidase (HRP)–conjugated goat anti-rabbit immunoglobulin G (IgG) secondary antibody (1:100; ab15285, Wuhan Boster Biological Technology Co., Ltd., Wuhan, Hubei, China) at 37°C for 1 h. The membrane was then immersed in enhanced chemiluminescence (ECL) solution (Pierce, Waltham, MA, U.S.A.) at room temperature for 1 min. At last, the membrane was exposed to a chemiluminescent gel imager (MicroChemi, ALIT Life Science Co., Ltd., Shanghai, China) for analysis for various parameters. The ratio of the gray value of the target band to GAPDH was representative of the relative protein expression.

### Monodansylcadaverine staining

The cells were inoculated into 24-well plates and incubated with corresponding drug interventions for 12 h. Subsequently, 50 μmol/l monodansylcadaverine (MDC) was added to the cells of each group for inoculation at 37°C with 5% CO_2_ for 1 h, which were then fixed with 2% paraformaldehyde. Next, the cells were permeated with 1% Triton X-100, stained with 4′, 6-diamidino-2-phenylindole (DAPI), sealed using an anti-fluorescence quencher and at last, observed under a laser confocal microscope.

### Flow cytometry

The cell apoptosis was measured via Annexin V-fluorescein isothiocyanate (FITC) and propidium iodide (PI) double staining (Sigma–Aldrich Chemical Company, St. Louis, MO, U.S.A.). In brief, the cells were incubated in a 37°C incubator with 5% CO_2_. After a 48-h period of culture, the cells were collected and centrifuged in 200 μl binding buffer. Next, a combination of 10 μl Annexin V-FITC and 5 μl PI was added to the cells to react for 15 min at room temperature devoid of light. Subsequently, 300 μl binding buffer was added, and the cell apoptosis was analyzed using an FACS Calibur flow cytometer (Becton, Dickinson and Company, Franklin Lakes, NJ, U.S.A.) at an excitation wavelength of 488 nm.

### Statistical analysis

Statistical analysis was conducted using the SPSS 21.0 statistical software (IBM Corp. Armonk, NY, U.S.A.). The test of normal distribution and homogeneity of variance was conducted on all specified dates. Measurement data in normal distribution were expressed as mean ± standard deviation. The comparisons between multiple groups were analyzed by one-way analysis of variance (ANOVA). A value of *P*<0.05 was considered to be of statistical significance.

## Results

### EPHA3 expresses poorly in endometriosis

**Figure 2:**
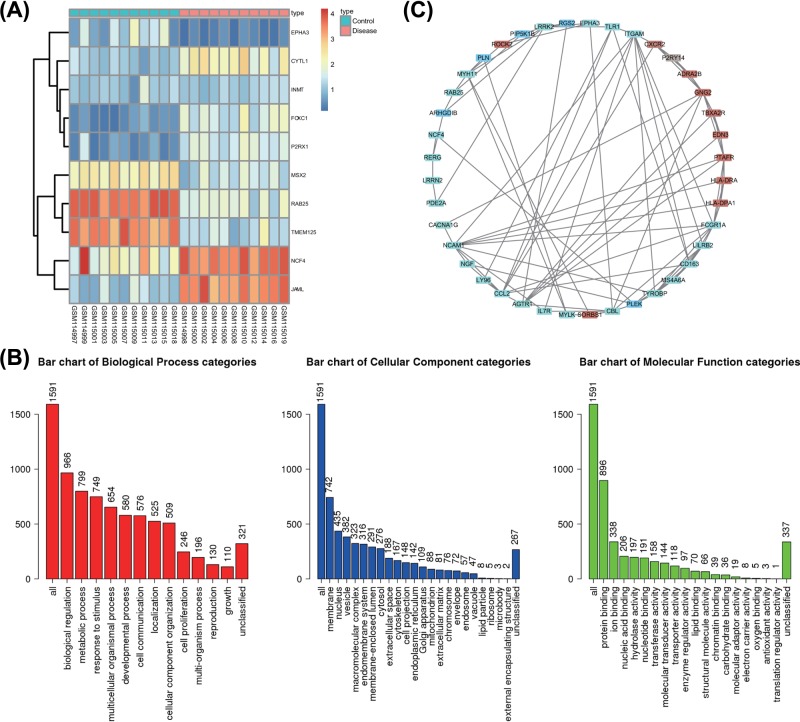
EPHA3 is elucidated to play a role in endometriosis progression (**A**) The heatmap of the expression of DEGs from the GSE5108 dataset; the abscissa represents the sample number, while the ordinate represents the gene name; the upper dendrogram indicates the sample type clustering, while the right histogram indicates the gene expression level; red indicates higher expression, while green indicates lower expression. Each rectangle corresponds to a sample expression value and the left dendrogram represents the clustering of gene expression. (**B**) GO analysis of DEGs. (**C**) The network map of co-expressed genes; the line indicates the interaction between two proteins, while blue indicates a low score for the combined score, and red indicates a high score for the combined score.

The gene expression dataset, GSE5108, was downloaded from the GEO database, and to screen out 1856 DEGs for our study. The literature has suggested that EPHA3 functions as a proangiogenic factor in multiple myelomas [23]. Moreover, a previous study highlighted the association of deletion of EPHA3 protein expression with advanced tumor-node-metastasis (TNM) in clear renal cell carcinoma [24]. Furthermore, hypoxia-controlled EPHA3 has been demonstrated to mark a multipotent mesenchymal stromal cell derived from human endometrium, which facilitates vascular growth [25]. However, the exact function of EPHA3 in human endometriosis remains unclear. The current study aimed at predicting the function and clinical significance of EPHA3 in endometriosis, from which EPHA3 ranked highly in DEGs. Figure 2A displays a heatmap of the DEGs in GSE5108, which exhibited a weakened expression of EPHA3 in endometriosis. Figure 2B displays the GO analysis of DEGs using the website (http://www.webgestalt.org/), which primarily focused on vital parameters such as biological process (BP), cellular component (CC) and molecular function (MF). Figure 2C showcases the PPI analysis of DEGs demonstrating that EPHA3 has a co-expression relationship with multiple genes. Furthermore, an existing study demonstrated that the abnormal activation of the mTOR signaling pathway stimulates cell proliferation, migration, invasion, and inhibits cell apoptosis in endometriosis [20,26].

### The mouse model of endometriosis is successfully established

**Figure 3:**
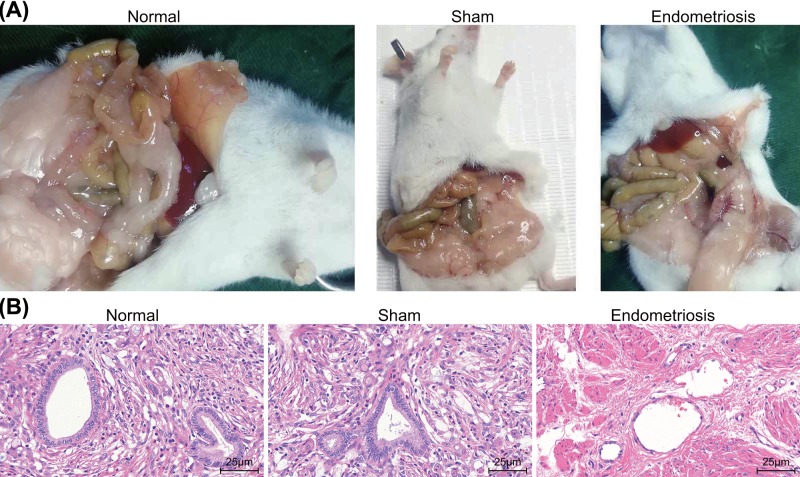
Successful mouse modeling of endometriosis is verified (**A**) The characteristics of endometriosis in each group of mice from visual inspection. (**B**) HE staining analysis of endometrial tissues in each group of mice (× 200).

After successful assessment of the expression pattern of EPHA3 in endometriosis, the focus of the experiment then shifted to establishing an endometriosis mouse model. First, visual inspection and HE staining were employed in an attempt to detect the key characteristics of endometriosis lesions in BALB/c mice of each group at the 21st day after induction. The results obtained from the visual inspection were as follows: no abnormalities were observed in the endometrial tissues of the mice in the normal group and the sham group, while the endometrial tissues of the endometriosis group presented with bright red ectopic lesions with different shapes (Figure 3A). The results obtained from HE staining manifested the presence of numerous glands in the endometrial epithelial cells of mice, where the glandular epithelial cells were mostly columnar, with vacuoles and apical secretion. In the endometriosis group, the endometrial epithelial cells of mice grew in a low-columnar ring or serrated shape with necrosis and inflammatory cell infiltration in some parts, and thinning of the endometrial stromal layer and fibrosis of different degrees were evident (Figure 3B). Collectively, the obtained results suggested a successful induction.

### EPHA3 is poorly expressed while mTOR is highly expressed in mice with endometriosis

**Figure 4:**
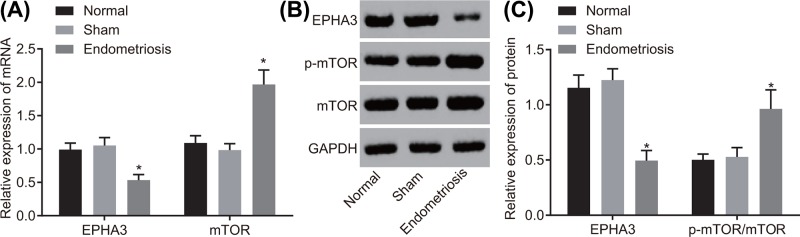
EPHA3 presents with a low expression while mTOR shows a high expression in mice with endometriosis (**A**) The mRNA levels of EPHA3 and mTOR in endometrial tissues of mice determined by RT-qPCR. (**B,C**) Western blot analysis of EPHA3 and mTOR proteins and the extent of mTOR phosphorylation in endometrial tissues of mice. *P<0.05 *vs.* the normal group; *n*=15; the data were analyzed using one-way ANOVA.

Reverse transcription quantitative polymerase chain reaction (RT-qPCR) and Western blot analysis methods were subsequently conducted in order to examine the mRNA (Figure 4A) and the protein (Figure 4B,C) levels of EPHA3 and mTOR in the endometrial tissues of mice. No significant difference was observed in the mRNA and protein levels of EPHA3 and mTOR and the ratio of p-mTOR/mTOR in endometrial tissues of mice in the normal group and the sham group (both *P*>0.05). On comparing with those of the normal group, it was observed that the mRNA and protein levels of EPHA3 in the endometrial tissues of mice in the endometriosis group significantly decreased, while the mRNA and protein levels of mTOR and the ratio of p-mTOR/mTOR increased (both *P*<0.05). These results implicated that EPHA3 was down-regulated while the mTOR signaling pathway was stimulated in mice with endometriosis.

### The mouse macrophages are successfully isolated

**Figure 5:**
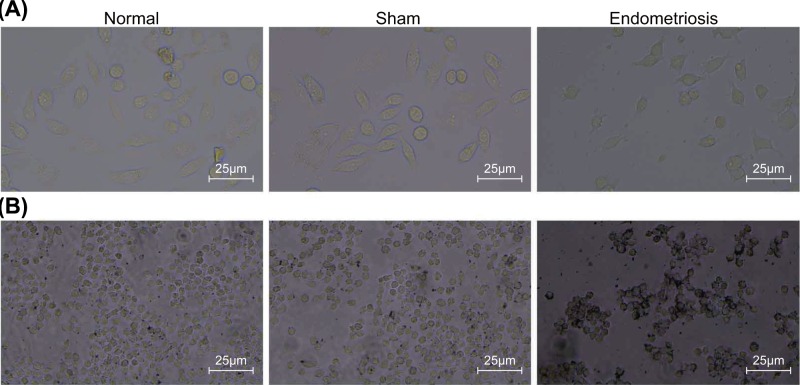
The macrophages are isolated through microscope examination and Trypan Blue staining (**A**) The morphological characteristics of macrophages after 24 h of isolation and culture observed under an inverted microscope (×400). (**B**) Trypan Blue staining analysis of survival rate of macrophages (×400). The experiment was repeated three times independently.

The changes of mouse macrophages were detected via microscopic examination and Trypan Blue staining. After culturing for 24 h, the results obtained from microscopic examination showed that the adherent cells of the normal group and the sham group were of different shapes such as round, oval, triangular or irregular, with pseudopod and protrusions. The cells were large in volume and the nucleus was kidney-shaped or oval, with abundant cytoplasm. In contrast, the morphology of the macrophages in the endometriosis group was smaller, with reduced cytoplasm and round boundaries (Figure 5A). Clear blue color on Trypan Blue staining was reflective of dead cells, while the living cells appeared colorless. In this experiment, the survival rate of macrophages in the normal group and the sham group was over 98%, while it was up to 92% in the endometriosis group (Figure 5B). Conjointly, the macrophages of mice were isolated through Trypan Blue staining.

### EPHA3 inhibits the activation of the mTOR signaling pathway

**Figure 6:**
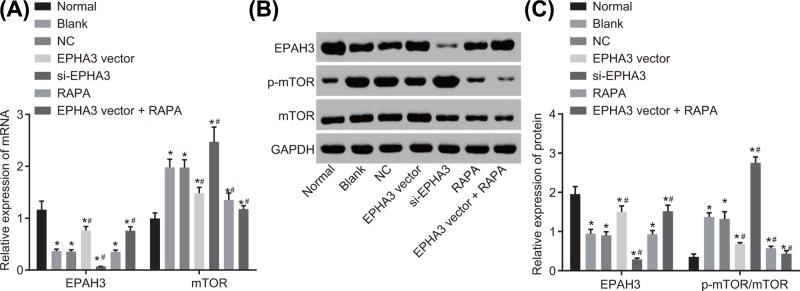
EPHA3 blocks the mTOR signaling pathway (**A**) The mRNA levels of EPHA3 and mTOR tested by RT-qPCR. (**B,C**) Western blot analysis of EPHA3 and mTOR proteins and the extent of mTOR phosphorylation in cells of mice after treatment. *P<0.05 *vs.* the normal group; #*P*<0.05 *vs.* the blank group and the NC group. The data in the figures were analyzed using one-way ANOVA.

The expression of EPHA3 and mTOR, as well as the extent of mTOR phosphorylation in macrophages of each group was evaluated by means of RT-qPCR (Figure 6A) and Western blot analysis methods (Figure 6B,C). The results displayed that compared with the normal group, the mRNA and protein levels of EPHA3 in the blank group decreased significantly, while the mRNA level of mTOR and the p-mTOR/mTOR increased (all *P*<0.05). No statistical difference was observed in the expression of EPHA3 and mTOR, as well as the extent of mTOR phosphorylation between the blank and NC groups (all *P*>0.05). When compared with those of the blank group and the NC group, the mRNA and protein levels of EPHA3 in the EPHA3 vector group increased significantly, while the mRNA level of mTOR and the ratio of p-mTOR/mTOR decreased significantly (all *P*<0.05). The mRNA and protein levels of EPHA3 in the si-EPHA3 group decreased significantly, whereas the mRNA level of mTOR and the ratio of p-mTOR/mTOR increased (all *P*<0.05). In contrast with the blank group and the NC group, the mRNA and protein levels of EPHA3 in the RAPA group did not depict significant alterations (*P*>0.05), while the mRNA level of mTOR and the ratio of p-mTOR/mTOR were significantly down-regulated (*P*<0.05). The mRNA and protein levels of EPHA3 in the EPHA3 vector + RAPA group were significantly up-regulated, and the mRNA level of mTOR and the ratio of p-mTOR/mTOR were notably down-regulated, the decline of which was more evident (all *P*<0.05). Collectively, these results denoted that EPHA3 inhibited the activation of the mTOR signaling pathway.

### EPHA3 promotes macrophage autophagy through mediating the mTOR signaling pathway

**Figure 7:**
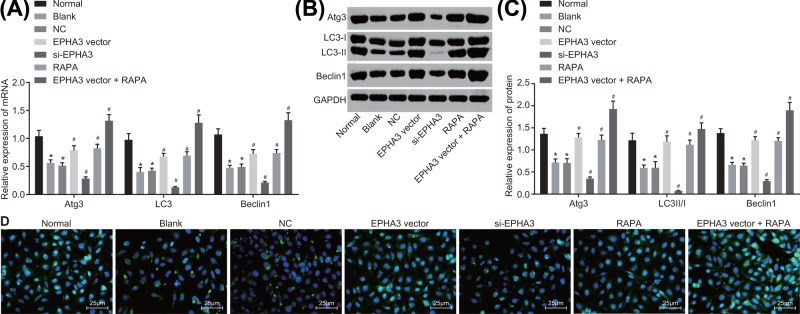
EPHA3 promotes the expression of Atg3, LC3, Beclin1 as well as the formation of autophagic lysosome (**A**) The mRNA levels of Atg3, LC3 and Beclin1 evaluated by RT-qPCR. (**B,C**) Western blot analysis of Atg3, LC3 and Beclin1 proteins. (**D**) MDC staining analysis of autophagic lysosome. *P<0.05 *vs.* the normal group; #*P*<0.05 *vs.* the blank group and the NC group. The experiment was repeated three times independently and data in the figures were analyzed using one-way ANOVA.

After examining which gene inhibits the activation of the mTOR signaling pathway, the succeeding objective in this experiment was to examine the underlying mechanism by which EPHA3 promoted macrophage autophagy through the mTOR signaling pathway. Initially, the RT-qPCR (Figure 7A) and Western blot analysis (Figure 7B,C) methods were performed in an attempt to measure the expression of autophagy-related genes. On comparing with the values of the normal group, it was observed that the mRNA and protein levels of Atg3, LC3 and Beclin 1 in the blank group decreased significantly (all *P*<0.05). Relative to the blank group and the NC group, mRNA and protein levels of Atg3, LC3 and Beclin 1 in the EPHA3 vector group, the RAPA group and the EPHA3 vector + RAPA group presented with elevated levels of the same, with the most significant changes observable in the EPHA3 vector + RAPA group (all *P*<0.05), while reverse trends were demonstrated in the si-EPHA3 group (all *P*<0.05).

The results obtained from MDC staining showed that when compared with that of the normal group, the number of autophagic lysosome was decreased in the blank group (*P*<0.05). In contrast with the blank and NC groups, the number of autophagic lysosome in the EPHA3 vector group, the RAPA group and the EPHA3 vector + RAPA group increased significantly, with evident enhancement in the EPHA3 vector + RAPA group (all *P*<0.05). The number of autophagic lysosome in the si-EPHA3 group reduced greatly (*P*<0.05) (Figure 7D). These results indicated that EPHA3 inhibited the activation of the mTOR signaling pathway and promoted macrophage autophagy of mice with endometriosis.

### EPHA3 promotes macrophage apoptosis through the inhibition of the mTOR signaling pathway

**Figure 8:**
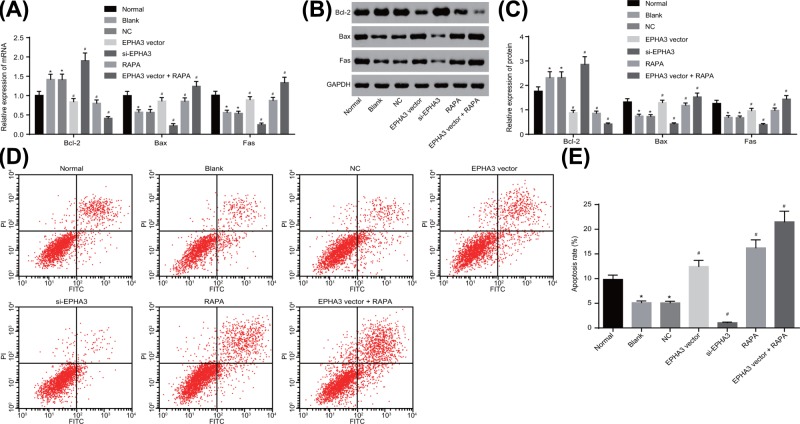
The macrophage apoptosis is promoted by EPHA3 up-regulation and blockade of the mTOR signaling pathway (**A**) mRNA levels of bcl-2, bax and fas in cells detected by RT-qPCR. (**B,C**) Western blot analysis of bcl-2, bax and fas proteins. (**D,E**) Macrophage apoptosis measured by flow cytometry. *P<0.05 *vs.* the normal group; #*P*<0.05 *vs.* the blank group and the NC group. The experiment was repeated three times independently. Data in the figure were analyzed using one-way ANOVA.

RT-qPCR (Figure 8A) and Western blot analysis (Figure 8B,C) methods were performed in order to measure the expression of apoptosis-related genes. These results indicated that compared with the values of the normal group, the mRNA and protein levels of bax and fas in cells of the blank group decreased significantly (both *P*<0.05), while the mRNA and protein levels of bcl-2 increased notably (*P*<0.05). No statistical difference was evident in the expression of apoptosis-related genes between the blank group and the NC group (all *P*>0.05). In comparison with the blank group and the NC group, the mRNA and protein levels of bax and fas in cells of the EPHA3 vector group, the RAPA group and the EPHA3 vector + RAPA group increased significantly (all *P*<0.05), while the mRNA and protein levels of bcl-2 were contradicting (*P*<0.05); in the si-EPHA3 group, the mRNA and protein levels of bax and fas decreased significantly (all *P*<0.05), while the mRNA and protein levels of bcl-2 were contradicting (*P*<0.05).

The results of flow cytometry for macrophage apoptosis demonstrated that the apoptosis rate of the macrophages in the blank group was significantly lower than that in the normal group (*P*<0.05). No statistical difference was observed in the apoptosis rate of the macrophages between the blank group and the NC group (*P*>0.05). In comparison with that of the blank group and the NC group, the apoptosis rate of macrophages in the EPHA3 vector group, the RAPA group and the EPHA3 vector + RAPA group significantly increased (all *P*<0.05), which significantly decreased in the si-EPHA3 group (*P*<0.05) (Figure 8D,E). The results demonstrated that EPHA3 suppressed the activation of the mTOR signaling pathway and promoted macrophage apoptosis.

## Discussion

Endometriosis is defined as a multifactorial, progressive fibrotic condition presenting itself in the form of uterine and surrounding stroma [27,28]. However, the lack of understanding of the mechanisms of endometriosis remains to be problematic. The present study showcased functionality of the EPHA3 as the suppressor of endometriosis via its disruption in autophagy and apoptosis of macrophages through inhibition of the mTOR signaling pathway.

The *in silico* analysis highlighted the ability of EPHA3 to suppress endometriosis via regulation of the mTOR signaling pathway. In addition, an important finding of the present study was that EPHA3 was poorly expressed, while the mTOR signaling pathway was activated in the endometrial tissues in mouse models with endometriosis. EPHA3 has the ability to function as an indicator in both kinase-dependent and kinase-independent manners, either by promoting or suppressing the tumor [29,30]. The tumor and stroma cells that are dependent on ligand and EPHA3 apoptosis indicated that wild-type EPHA3 was resistant to the tumor in non-small-cell lung carcinoma (NSCLC) [31]. Additionally, in the current study, a negative correlation was evident between the mTOR signaling pathway and EPHA3, with emphasis on the role as a mediator in the pathogenesis of endometriosis. Existing evidence has highlighted functionality of the PI3K/Akt/mTOR signaling pathway as a modulator of proliferation, which aids in the survival of endometriosis cells [20]. In addition to its aid in the survival of the endometriosis cells, an investigation conducted to examine the relationship between EPHA3 and the expression of signaling proteins flagged the suppression of several signaling proteins, including PI3K, STAT3 and BMX as a result of EPHA3 overexpression [31]. Furthermore, the activation of mTOR signaling pathway has been proven to be beneficial to patients suffering from endometrial cancer [32]. Therefore, it was concluded that EPHA3 is capable of functioning as an inhibitor for the activation of the mTOR signaling pathway.

Another vital finding in this experiment showcases the advantageous properties of EPHA3 in the autophagy of macrophages in endometriosis. Autophagy is defined as the catabolic process related to the degradation and removal of both damaged organelles and protein aggregates [33]. Defined as important components of the acting autophagic machinery, LC3 and Atg3 present an abnormal expression in autophagy [34]. Another autophagy protein, Beclin 1, which is a part of the class III PI3K complex, can induce autophagy, and provide evidence supporting the importance of the decomposition of Beclin 1 from Bcl-2 in autophagy [35]. Therefore, the observation of increased expression of Atg3, LC3 and Beclin 1 in this study elucidated the ability of the EPHA3 as an activator of macrophage autophagy. Autophagy and apoptosis usually emerge in the same cell, and in most cases, the former precedes the latter [36]. Both the bax and fas genes function as significant regulatory genes for the progression of cell apoptosis, which present with increased expression in some solid cancer cells [37]. Along with the increased expression in some cancer cells, the anti-apoptotic member of the bcl-2 family proteins, bcl-2, could critically mediate intrinsic apoptosis, suppressing the cell death, which present with low expression to the treatment for many cancers [38]. Also, activation of the EPHA3þ/CD90þ/Sca1þ mesenchymal/stromal cells with an EPHA3 agonist stimulates cell contraction, cell–cell segregation and apoptosis [39]. Therefore, through increased expression of bax and fas and decreased expression of bcl-2 in this study, we hypothesized that EPHA3 promoted the apoptosis of macrophages, with evidence supporting the role of EPHA3 as a potential therapeutic target for the treatment of endometriosis.

In conclusion, the evidence collected from our study supports the contribution of the EPHA3 in the treatment of patients with endometriosis by means of its promoting effects on macrophage autophagy and apoptosis through inactivation of the mTOR signaling pathway (Figure 1B). Taken conjointly, it is suggested that EPHA3 holds potential as a therapeutic target for the treatment of patients with endometriosis. However, the related report on the function of EPHA3 in endometriosis progression is still under development, and further assessments focused on elucidating the underlying mechanism of EPHA3 in endometriosis are required.

## Abbreviations

AKT: protein kinase B
Atg3: autophagy-related gene 3
bax: bcl-2 associated X protein
bcl-2: B-cell lymphoma*-*2
BMX: BMX non-receptor tyrosine kinase
DEG: differentially expressed gene
EPHA3: erythropoietin-producing hepatocellular carcinoma A3
FITC: fluorescein isothiocyanate
GAPDH: glyceraldehyde-3-phosphate dehydrogenase
GEO: Gene Expression Omnibus
GO: gene ontology
HE: Hematoxylin–Eosin
LC3: light chain 3
MDC: monodansylcadaverine
mTOR: mammalian target of rapamycin
NC: negative control
PI: propidium iodide
PI3K: phosphatidylinositol 3-kinase
PPI: protein–protein interaction
RAPA: rapamycin
RT-qPCR: reverse transcription quantitative polymerase chain reaction
